# *Scenedesnus rotundus* isolated from the petroleum effluent employs alternate mechanisms of tolerance to elevated levels of Cadmium and Zinc

**DOI:** 10.1038/s41598-019-44374-1

**Published:** 2019-06-11

**Authors:** Subhashini Shivaji, Sarada V. L. Dronamaraju

**Affiliations:** Department of Biotechnology, School of Bioengineering, SRM Institute of Science and Technology, Chennai, India

**Keywords:** Plant physiology, Abiotic

## Abstract

*Scenedesmus rotundus* was isolated from metal contaminated petroleum industry effluent and its tolerance to Cadmium and Zinc was tested using different concentrations of CdCl_2_ and ZnCl_2_ ranging from 0.001 mM to 1.0 mM of Cd and 0.03 mM to 1.21 mM of Zn amended in Bolds Basal medium. The changes in cell count recorded at regular intervals upto a period of 24 days revealed a concentration dependent inhibition in growth. Concentration of the metal, at which 50% of the cells are live and metabolically active referred to as EC_50_ was calculated as 0.04 mM for Cd and 0.2 mM for Zn. Further, the effect of EC_50_ of the metals on the protein content, uptake of metals at varying pH, oxidative stress markers including lipid peroxidation, protein oxidation andnd oxygen uptake, levels of enzymatic antioxidants such as catalase and superoxide dismutase and non-enzymatic antioxidants namely, GSH and PC_4_ were determined. Though a direct correlation could not be drawn between pH and metal uptake, the compartmentalization of the metal during the lag phase and exponential phase was evident, most of the metal was present in extracellular fractions in the former, while in the later it was internalized. Our study shows a clear correlation between toxicity of Cd and the ability of the algae to synthesize PC_4_ from GSH and chelate it leading to detoxification, while Zn treatment led to an increase in the activity of catalase and superoxide dismutase and replete GSH pools. Further the changes in the cell wall structure at EC_50_ of Cd and Zn were studied. This is the first report on effect of heavy metals on the structural modifications of the cell wall of *Scenedesmus* in general and *Scenedesmus rotundus* in particular, indicating appearance of granules on the entire cell surface in both Cd and Zn treatments, with the degree of granulation increasing in the order of pH 12 > 10 > 8 in Cd treatment. Further structures of higher order resembling minute wheels are observed in Cd treated cells are also reported.

## Introduction

Several transition metals like Cu, Fe and Zn have acquired metabolically indispensable roles due to their chemical and physiological properties^1^. Redox-active metal ions can trigger the formation of hydroxyl radicals^2^ leading to oxidative stress. Due to various anthropogenic activities levels of heavy metals in the environment has drastically increased. Organic molecules and hydrogen ions in the aquatic systems influence metal speciation^3^, leading to improved bioavailability and increased metal toxicity^4–7^. The affinity of the biological molecules with sulphur, nitrogen and oxygen containing functional groups to heavy metals leads to inactivation and shutting down of vital biological activities. This necessitates activation of detoxification mechanisms to overcome the fluctuation concentration of metal ions and to maintain homeostasis^8^.

Metal toxicity towards organisms is determined by the growth inhibition. To understand the toxic effects of metals, partitioning between the cell surface and medium is an important parameter affecting the survival of the organisms. The concentration of the ions is the most important parameter in the absorption processes. pH of solution influences the solubility of the metal, active groups on the cell surface and nature of binding sites on the algal cells. The attachment of metal to the external structures of the organism and subsequent uptake depends on the degree of protonation and the displacement of the protons from surface biomolecules^9–11^. Majidi *et al*.^12^, demonstrated that though acidic pH did not alter chemical nature of the binding sites it favoured the exchange of Cadmium in *Stichococcus bacillaris*.

Water pH is one of the important criteria for the survival of organism and the uptake of the metals by the organism. Several studies have shown the influence of complexation and pH on either individual or combined heavy metal toxicity to green algae^13^. While some studies have proved increases metal toxicity at low pH^14,15^. due to the presence of higher amount of free metal ion^14,16,17^, others showed that the metals like Cadmium, Copper and Zinc have a decreased the toxic effect at low pH^18–20^. A recent study has shown that acidification affects the metal availability and algal uptake not only by proton inhibition but also altered cell permeability^21^.

Metal biosorption has frequently been shown to be strongly pH dependent in almost all systems examined, including bacteria, Cyanobacteria, algae, and fungi. Competition between cations and protons for binding sites means that biosorption of metals like Cu, Cd, Ni, Co and Zn is often reduced at low pH values^22,23^. The cell wall being the first structural defence component helps protect the protoplast against metal toxicity. Previous studies report adsorption of metal ions to isolated algal or bacterial cell wall but not on the intact walls of living cells^24^.

There is huge area of study on algae and its use for bioremediation, however most of these studies are on the dead biomass^25^. Growth of living cells improves biomass thus increasing the metal absorption capacity. Further living cells also have a greater capacity for active and passive uptake mechanisms of heavy metal uptake, while only passive sorption occurs on dead material. Hence, factors affecting growth and physiology of the algae should first be investigated for the purpose of bioremediation. Among these, assessment of the inhibitory effects of metal concentrations is critical.

Formation of Reactive Oxygen species (ROS), such as superoxide anion (O2−), hydrogen peroxide (H_2_O_2_), singlet oxygen and the hydroxyl radical (OH−), occurs in all aerobic organisms as byproducts of oxidative metabolism. Some of them may function as important signaling molecules at low concentrations, at high concentrations all ROS can be extremely harmful, causing protein oxidation, lipid peroxidation, and damaging nucleic acids, eventually causing alterations in cell structure and mutagenesis^26,27^. Ascorbate and glutathione are part of a highly complex and intricate plant antioxidative system^28^. They function alongside catalases peroxidises and dismutases in high capacity redox- homeostatic H_2_O_2_-processing pathways. In addition GSH in eukaryotes plays a vital role in other physiological functions like conjugation of metabolites, and detoxification of xenobiotics^29,30^. This has been reported inseveral species including *Dunaliella tertiolecta*^31^ in response to zinc (Zn^2+^), *Chlamydomonas acidophila*^32^ treated with Cd^2+^, and marine diatoms: *Phaeodactylum tricornutum*^33^ and *Ceratoneis closterium*^33^ under Cu^2+^ stress.

In response to the elevated metal concentrations various protective mechanisms involving the synthesis of GSH (Glutathione) and amino acids such as Cysteine^34^ and synthesis of metal binding oligopeptides referred to as PCs (Phytochelatine) are reported^35,36^. These are oligomerized from GSH with activity of Phytochelatin synthase. Heavy metal complexation with cysteine residues in PC leads to the formation of PC-Metal complex through thiol coordination followed by sequestration and subsequent detoxification^37^.

Apart from the internal metabolite synthesis the cells also show external morphological differences. Various studies have been reported physiological and morphological changes as responses to heavy metal detoxification. Differences in metal tolerance among species, or among strains of the same species are often encountered and haveoften been attributed to changes in cell wall architecture. The capacity of the cell wall to bind metal species improves tolerance to heavy metals, as reports indicate that tolerant species to bind more metal to the cell wall, than the comparable less tolerant strain. Algal cell wall has the capacity to bind metal ions with its negatively charged sites^38,39^, polysaccharides^10^, and certain unprotonated groups such as a carboxyl oxygen or sulphate^40^.

The growth kinetics of the algae, measure the tolerance/ability of the organism to survive in the particular environment. In this study we examined the tolerance levels of *Scenedesmus rotundus*, a species isolated from petroleum industry effluent to elevated levels of Cd and Zn based on growth measurements and variations in pigment content. EC_50_ concentrations i, e the limit where metal uptake kinetics and growth are balanced under non-complexing medium conditions were analysed. Further the effect of varying pH (5–12) on the uptake of Zn and Cd by the selected strains was studied at EC_50_ concentrations of Cd and Zn in terms of surface-bound, (adsorbed and absorbed) and intracellular metal fractions. Tolerance mechanisms were understood by analysing the enzymatic and non-enzymatic anti-oxidants and detoxification. In addition the ultra-structural modifications related to surface morphology of the alga challenged with metal were also studied.

## Materials and Methods

### Isolation and growth of *Scenedesmus rotundus*

Microalgal species were isolated from the effluent discharged from fertilizer factories in and around Chennai, India. Unialgal isolates strains were obtained by repeating sub-culturing according to the method described by^41^ on Bolds Basal Medium at 25 °C ± 2 °C continuously illuminated with an incident light of 50 μE/m^2^/s, photoperiod 16–8 h on a gyratory shaker at a speed of 130 rpm/min and regularly sub cultured until use. Theorganism was grown under the above mentioned conditions for all experiments.

### Heavy metal tolerance

The isolated algae were seeded at 10^4^ cells/ mL, in Bold’s Basal Medium amended with 0.001, 0.01, 0.1, 1.0 mM concentrations of Cadmium Chloride, with medium without Cadmium serving as control and 0.09, 0.18, 0.27, 0.81, 1.21 mM concentrations of Zinc Chloride, where 0.03 mM, the native concentration of Zn in medium served as control.

### Growth

Growth of the isolated algal strains was monitored by recording the number of cells using a hemocytometer, at 4 day interval up to a period of 24 days and expressed as total no. of cells per mL of the culture medium. Concentration at which 50% of the cells were live and metabolically active (EC_50_) was determined for both the metals based on the cell count using probit analysis.

### Estimation of chlorophyll

For analyses of pigments, 5 ml of the algal culture was drawn, cells were pelleted and the pellet was suspended in 80% acetone for 24 h at 4 °C. The suspension was centrifuged and the supernatant was read at 470, 645 and 663 nm and the OD values were used for estimation of chlorophyll a, chlorophyll b and carotenoid according to^42^.

### Estimation of protein

One mL of algal sample was centrifuged 5000 rpm for 5 min. Supernatants were collected for the protein assay and the pellet was re-extracted with 1.0 mL of 0.1 N NaOH with 0.5% β-Mercaptoetanol (v/v). The mixture of NaOH and pellets were incubated at room temperature for 1 hr and then centrifuged at 5000 rpm for 10 mins. The supernatant was combined with first and pellet was discarded. The final volume of the extract was 2.0 mL the proteins was estimated by following the method of Lowry *et al*.^43^, Bovine Serum Albumin was taken as the standard. All the experiments were conducted in triplicates and values expressed as mean ± SD.

### Effect of pH on growth and uptake of cadmium and zinc

The algal cells were grown in Bold’s Basal Medium amended at EC_50_ concentrations of Cd and Zn at different pH ranging from 5–12, (5, 8, 10 1nd 12) with native pH of the medium 6.8 serving as control. Cells were harvested on Day 4 and Day 12. A modified method of Franklin *et al*.^44^ was used to determine intracellular and extracellular concentrations of Cd and Zn. Algal cells were harvested by centrifugation and the supernatant representing the soluble metal fraction was referred to as fraction F1. The pellet was rinsed thoroughly with 2 mL Bold’s Basal media and centrifuged. The supernatant representing the concentration of metal absorbed to the cells was referred to as fraction F2. The pellet was washed with 2 mL of EDTA solution (0.01 M EDTA, 0.1 M K_2_H_2_PO_4_ pH 6) and centrifuged, the resulting supernatant referred to as F3 represented the concentration of the metal adsorbed on to the surface of the alga. The algal cell pellet was digested in 2 mL concentrated Nitric acid for 10 hours and the fraction representing intracellular metal concentration was referred to as Fraction F4. The culture flasks in which the algal cells were grown were rinsed with 10% HNO_3_ representing the concentration of the metal absorbed to the glass was referred to as fraction F5. All the fractions were analysed using atomic absorption spectrometer (Element AS AAS4141, (Electronic Corporation of India) and the metal concentrations calculated against standard CdCl_2_ and ZnCl_2_ solutions.

### Effect of Cd and Zn on the levels of oxidative stress markers

#### Lipid peroxidation

Algal extract (1 mL) was taken in a 10 mL test tube and mixed with 1 ml of TBA. The mixture was heated in a boiling water bath at 95 °C for 60 minutes. The test tubes were cooled at room temperature and absorbance was measured at 532 nm using UV-visible spectrophotometer.

#### Protein oxidation

Carbonyl derivatization reaction with DNPH was performedaccording to Levine *et al*.^45^ with some modifications. To 5 mL of algal cell extract, 18% SDS was added and mixed. Equal volume of 20 mM DNPH in 20%trifluoroacetic acid (TFA) was added followed by 20 mL of 2 M Tris in 30% glycerolwith 0.74 M β-mercaptoethanol. The samples were incubated at 37 °C for 30 min. Samples without DNPH served as control. The absorbance of thesupernatant was measured at 375 nm

#### Oxygen uptake

Oxygen uptake was measured by oxygen electrodes withcomputerized data acquisition described byBaker *et al*.^46^. The algal cells were shaken inbeakers to ensure continuous aeration of the media. Changes in the steady state concentration of oxygen inbeakers containing algal cells was compared to beakers with medium alone (100%) to estimate the basal rate of respiration.

### Effect of Cd and Zn on the activity of enzymatic antioxidants

The algal cells were centrifuged and homogenized with silica beads in 2-ml vials by application of two times 60-s cycle. The homogenised sample was resuspended in the Bolds Basal Medium used for enzyme assays.

#### Superoxide dismutase (SOD)

SOD was estimated as per the procedure described by Kakkar *et al*.^47^. Algal homogenate (0.5 ml) was diluted to 1 mL with 0.5 mL of distilled water. To the suspension 0.25 mL of ice coldethanol and 0.15 mL chloroform were added. The mixture was shaken for 1 minute and centrifuged at 2000 rpm. To the supernatant, 1.5 ml of carbonate buffer was added. The reaction was initiated by the addition of 0.4 ml epinephrine and change in optical density per minute was measured at 480 nm in a double beam UV-VIS spectrophotometer (UV 1700, Szhimadzu). SOD activity was expressed as U/mg of protein. Change in optical density per minute at 50% inhibition to adrenochrome transition by the enzyme is taken as one enzyme unit.

#### Catalase

Catalase activity was assayed by the method of Sinha *et al*.^48^. To 0.1 ml algal homogenate, 1.0 ml eachof phosphate buffer and hydrogen peroxide were added. The reaction was arrested by the addition of 0.2 ml dichromate acetic acid reagent. Standard hydrogen peroxide in the range of 4 to 20 µl was taken and treated similarly. The tubes were heated in a boiling water bath for 10 min and read at 570 nm in a double beam UV-VIS spectrophotometer (UV 1700, Szhimadzu). Catalase activity was expressed as U/mg protein.

#### Ascorbate peroxidase

Ascorbate peroxidase (APX) activity was assayed following Nakano and Asada^49^. The reaction mixture consisted of 100 μL ascorbate (7.5 mM), 100 μL algal extract, 100 μL H_2_O_2_(300 mM) and 2.7 mL of 25 mM potassium phosphate buffer with 2mMEDTA (pH7.0). Change in absorbance at 290 nm (ε = 2.8 mMcm^−1^) was considered as a measure of oxidation of ascorbate. Ascorbate peroxidase activity was expressed as U/mg protein.

### Effect of Cd and Zn on algal thiol content (GSH, and PCn)

Algal growth was followed for 12 days, corresponding to the time for the algae to reach the end of the exponential phase. One mL aliquots were taken at regular time intervals and cell counts were determined by haemocytometer. The algal cells were harvested at the density of 6 × 10^6^ cells/ml and centrifuged (3000 × *g* for 10 min at 20 °C). The preparation of the sample and derivatization steps were followed from^50^.

#### Extraction of GSH and PCs

The algae were filtered onto 0.8 µm Membrane and placed in 2 ml of 50 mM HEPES and 50 mM NaCl (pH 7.5) containing 1 mM TCEP. The cells were homogenised, centrifuged and supernatant filtered through 0.2 µm membrane.

#### Derivatization of GSH and PCs with mBBr

The extracted sample was dissolved in 300 µl of 6.3 mM DTPA with 0.1% (v/v) TFA. The derivatization protocol was followed from F. Else C. Sneller 2000^51^. 450 µl of 200 mM HEPES buffer, pH 8.2, containing 6.3 mM DTPA was mixed with 10 ml of 25 mM mBBr. Derivatization was carried out for 30 mins at 45 °C in the dark. The reaction was stopped by adding 300 µl of 1 M MSA. The samples were stored in the dark at 4 °C until HPLC analysis. Standard GSH PC3 and PC4 were also derivatized following the same procedure.

#### HPLC analysis

PCs were separated on Agilent 1260 infinity analytical column. Before injection, the column was equilibrated in methanol and water, the derivatized sample was injected and run in a 12–25% (v/v) methanol gradient for15min and then with a linear gradient ethanol from 15 to 29 min. Next, a linear gradient was used from 35% to 50% (v/v) methanol, from 29 to 50 min after injection. Fluorescence was monitored with an excitation wavelength of 380 nm and emission wavelength of 470.

### Effect of Cd and Zn on algal cell wall morphology

*Scenedesmus rotundus* was grown in Bolds Basal medium at varying pH amended with EC 50 concentrations of Cd and Zn. During the exponential growth phase the algal cells were centrifuged, the pellet was washed with phosphate buffer saline for 3 times. 2.5% glutaraldehyde was added and incubated at room temperature for 30 minutes. The pellet was washed with Na-phosphate buffer for 3 times and the pellets were collected. The sample was dehydrated with 30%, 50%, 70%, 80%, 90% ethanol for 10 minutes each and at 100% ethanol for 1 h. The structural changes were examined using Scanning Electron Microscope (Quanta 200 FEG)

### Statistical analysis

All the experiments were performed in three biological and three technical replicates. Results were expressed as mean ± SD for six replicates. All the data were statistically evaluated with SPSS/19.0 software.

## Results and Discussion

### Isolation and growth of *Scenedesmus rotundus*

**Figure 1:**
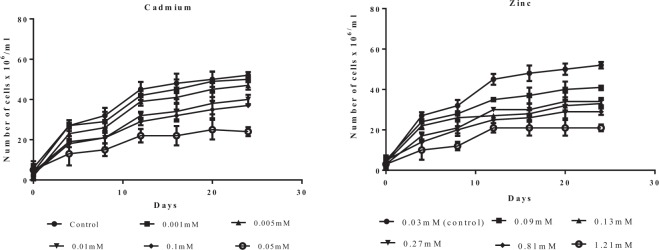
Effect of different concentrations of Cadmium (CdCl_2_) and Zinc (ZnCl_2_) on the growth of *Scenedesmus rotundus* isolated from metal contaminated water sample.

Based on the morphology and 18S rDNA sequence data, the species was identified as *Scenedesmus rotundus*. The effects of different concentrations of Cadmium and Zinc on the growth of *Scenedesmusrotundus* are presented in Fig. 1. Algal growth rate was affected by exposure to Cadmium and Zinc at higher concentrations. Among the different concentrations of Cadmium studied, growth of the alga was comparable to control, up to a concentration of 0.05 mM, at higher concentrations a rapid decline in growth was observed. Zn treated cultures of *Scenedesmusrotundus* tolerated the heavy metal upto the highest concentration of 1.21 mM studied, however a dose dependent reduction in growth was observed.

Based on the results obtained from the growth of *Scenedesmus rotundus* at varying concentrations of Cadmium and Zinc, EC_50,_ values, i, e concentration of the metals at which 50% of the cells are live and metabolically active was calculated. EC_50_ for Cadmium and Zinc was calculated to be 0.04 mM and 0.2 mM respectively. Previous studies have shown varying tolerance of different *Scenedesmus* species to elevated levels of Cadmium and Zinc. Baścik-Remisiewicz^52^ reported EC_50_ for Cd (18 mg/L)in *Scenedesmus armatus*, while the value was 810 mg/L in *S. rubescence* for Zn, and while EC_50_ for Zn in *S. acuminatus* was only 394 μg L^−1^. Magdaleno *et al*.^53^, and Monterio *et al*.^54^, reported *S. obliquus* tolerated Cd and Zn with EC_50_ values of 0.058 mg/L for Cd and 16.99 mg/L for Zn. As the alga was isolated from metal contaminated effluent containing 0.05 mg/L Cadmium and 0.12 mg/L Zinc it showed tolerance to both Cd and Zn with an ability to grow in a medium amended with 0.05 mM Cd and 1.21 mM Zinc.

### Effect of Cd and Zn on total chlorophyll

**Figure 2:**
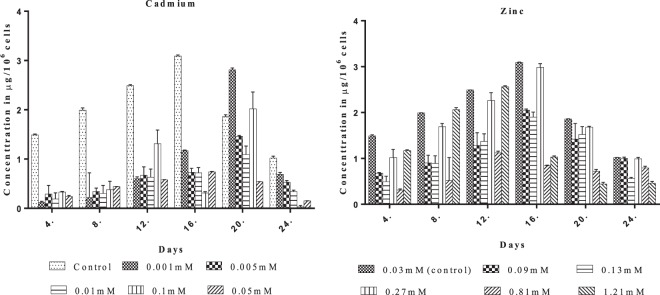
Effect on of different concentrations of Cadmium (CdCl_2_) and Zinc (ZnCl_2_) on the total Chlorophyll content of *Scenedesmus rotundus*.

Alterations in the pigment content are generally considered as a measure of physiological competence. Exposure of algal cells to metals above their tolerance limits results in reduction in the pigment content indicating bulk metabolic perturbations. In the present study the total chlorophyll of *Scenedesmus rotundus* showed significant variations when compared to the control in both Cd and Zn treatments (Fig. 2). Treatment with Cd caused a marked reduction in the total chlorophyll content across all the treatments studied, these results are in line with the findings of^55^.

### Effect of EC_50_ concentrations of Cd and Zn on the total protein content

**Figure 3:**
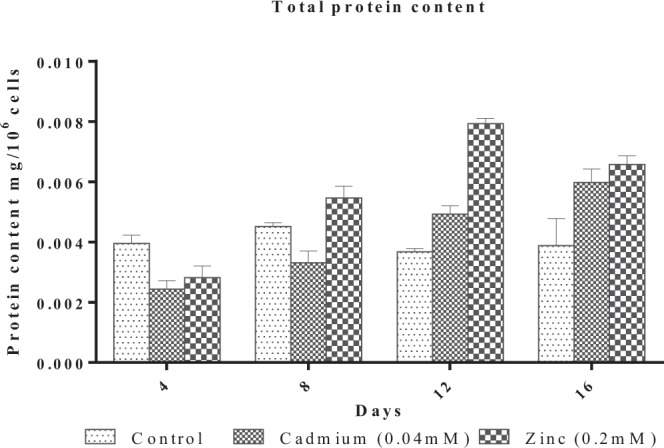
Effect of EC50 Concentrations of the Cadmium (CdCl_2_) and Zinc (ZnCl_2_) on the total protein content of *Scenedesmus rotundus*.

Protein content in algal cells is influenced by metal toxicity. *Scenedesmusrotundus*the total protein content was less in Zinc and Cadmium during the lag phasewhen compared to the control gradually increased in the exponential phase (Fig. 3). Among the two metals studied, Zinc treatment shows the higher protein contentwhen compared to the Cadmium. The increase of soluble protein is attributedto a variety of metabolic processes in cells, heavy metal stress can induce related stress protein gene expression, which is a defense mechanism of plants to environment stress^56^. Some reports indicate increased protein content in algae under metal stress, a decrease of soluble protein content was detected in Cdtreated *C. sorokiniana* cells (REF). Probably, chlorophylls and proteins, and even the chloroplast proteins, represented an emergency source of nitrogen and sulphur to ensure cell growth.

### Effect of pH on the uptake and internalization of cadmium and zinc

pH may affect the uptake of Zn and Cd by phytoplankton in water bodies by three possible mechanisms (1) the kinetic parameters of the Zn and Cd uptake systems may be directly affected; (2) the uptake systems for Zn and Cd may be up- or down-regulated; and (3) the bioavailability of the metals may change the localization of Cd and Zn in the intracellular compartments during exponential phase is attributed to the regulatory mechanisms operating within the cells.

**Figure 4:**
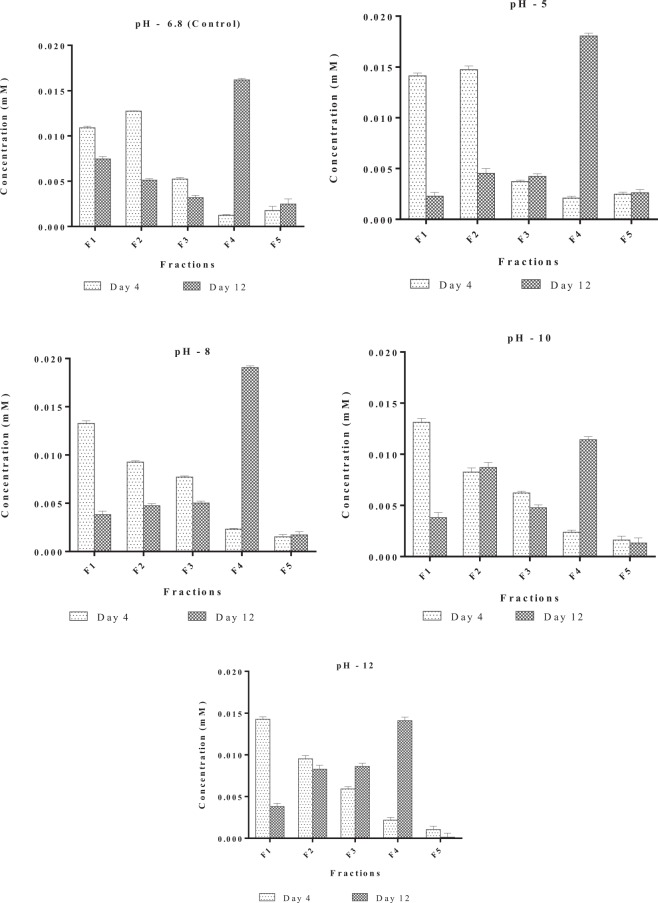
Effect of pH on internalization of Cadmium (CdCl_2_) in *Scenedesmus rotundus*. F1- soluble fraction, F2 - Fraction adsorbed, F3 - Absorbed fraction, F4 - Intracellular fraction, F5 - Fraction adsorbed to glass.

**Figure 5:**
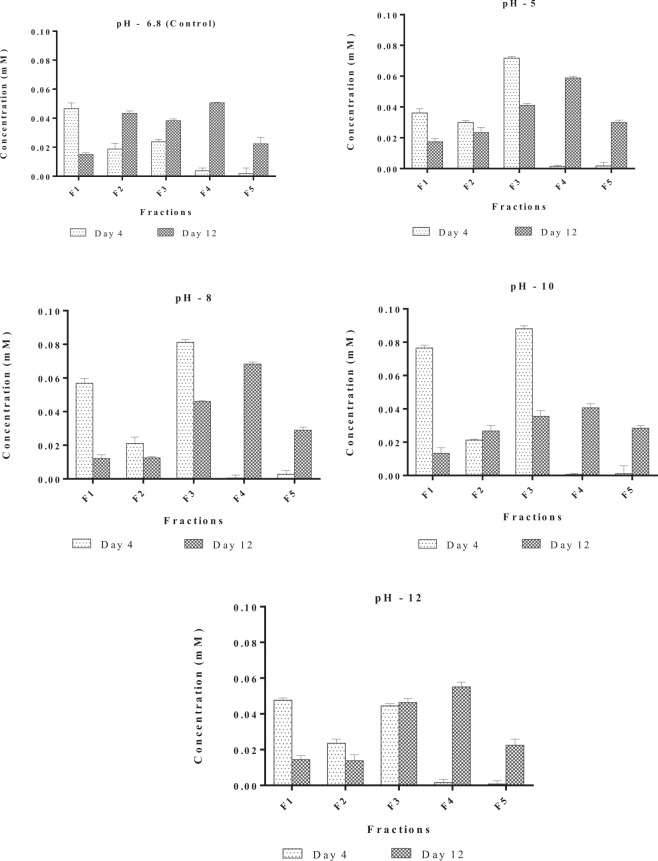
Effect of pH on internalization of Zinc (ZnCl2) in *Scenedesmus rotundus* F1- Soluble fraction, F2 - Adsorbed fraction, F3 - Adsorbed fraction, F4 - Intracellular fraction, F5 - Fraction adsorbed to flask.

Concentration of Cd and Zn were estimated in different fractions on Day 4 and Day 12 at EC_50_ concentrations of Cd and Zn at varying pH, pH 5, pH 6.8, (Control), pH 8, pH 10 and pH 12. Figures 4 and 5 depict the concentration of Cd and Zn in different fractions. During the lag phase of growth (Day 4) most of Cd was distributed between the soluble, adsorbed and absorbed fractions, while Zn was distributed between soluble and absorbed fractions. On the contrary, during exponential phase (Day 12) a large fraction of Cd was present in the intracellular fraction, while Zn was distributed between the absorbed and intracellular fractions. A direct correlation between the effect of pH and metal uptake by the alga was not observed in the present study. Similar observations were also reported by^57,58^. This could probably result due to buffering activity of numerous acid base groups, organic moieties and cell surface hydroxyls. Variation in pH in the bulk medium should be greatly attenuated at the surface of the cells as it is at particle surfaces with acid–base functionalities^59^.

### Effect of Cd and Zn on the activity of enzymatic antioxidants

**Figure 6:**
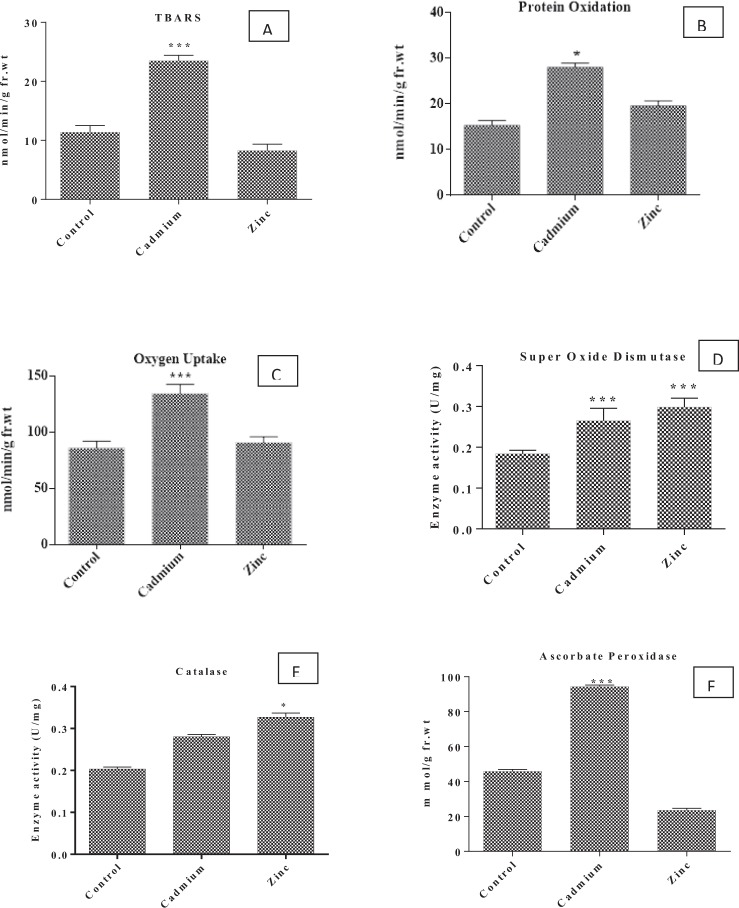
Effect of EC50 Concentration of Cadmium and Zinc on the levels of oxidative stress markers TBARS (**A**), Protein Oxidation (**B**), Oxygen Uptake. (**C**) and enzymatic antioxidants Superoxide Dismutase (**D**), Catalase (**C**), and Ascorbate peroxidase (**F**). Values represented as Mean ± SD of three replicate (***p < 0.005, *p < 0.05).

Synthesis of enzymatic and non-enzymatic anti-oxidants is a common response encountered in algae upon exposure to heavy metals. Enzymatic antioxidants such as such as superoxide dismutase, catalase, and ascorbate peroxidase and low molecular weight compounds non-enzymatic antioxidants such as carotenoids and glutathione often are involved in scavenging ROS. Damage to the cells often results Catalase and Super oxide dismutase activities are frequently reported to increase due to heavy metal stress^55^. Oxidative stress markers, lipid peroxidation, and oxygen uptake showed significant increase in Cd treated cells, while protein oxidation was significant in Zn treated cells when compared to the control. In the present study activity of catalase and super oxide dismutase increased with respect to control, a significant increase was recorded in Zn treated cultures when compared to Cd treated cultures, while ascorbate peroxidise activity showed significant increase in Cd treatment as compared to Zn treated cultures (Fig. 6).

### Effect of Cd and Zn on algal thiol content (GSH, and PCn)

As a protective measure, cells also induce the synthesis of protective proteins. These can be either metal chelators or enzymatic or non-enzymatic antioxidants or both. Metal chelators such as PCs^60–64^ are cysteine-rich peptides with an ability to coordinate metals to sulfhydryl groups on the protein. Phytochelatin synthesis appears to be the central point in the stress response to heavy metals in almost all the species studied. Most reports indicate induction of the phytochelatin synthase or an increase in the levels of PCs in the cells when exposed to heavy metals in a concentration-dependent and dose-dependent manner. Several types of PCsare reported ranging from PC_2_ to PC_4_ with respect to the metal studied, the concentration and the duration of exposure.

The response to heavy metal stress in *Scenedesmu*s critically influenced by the thiol GSH, and phytochelatins. Several independent studies corroborate the fact that these components are involved in the cells’ antioxidant metabolism to confer resistance to the heavy metal by efficient sequestration of the metal ions. Different metals tend to induce different patterns of regulation of the above said components. The most extensively studied is Cadmium, which has been shown by several independent studies to increase the GSH and phytochelatin content in the cell^65,66^.

**Figure 7:**
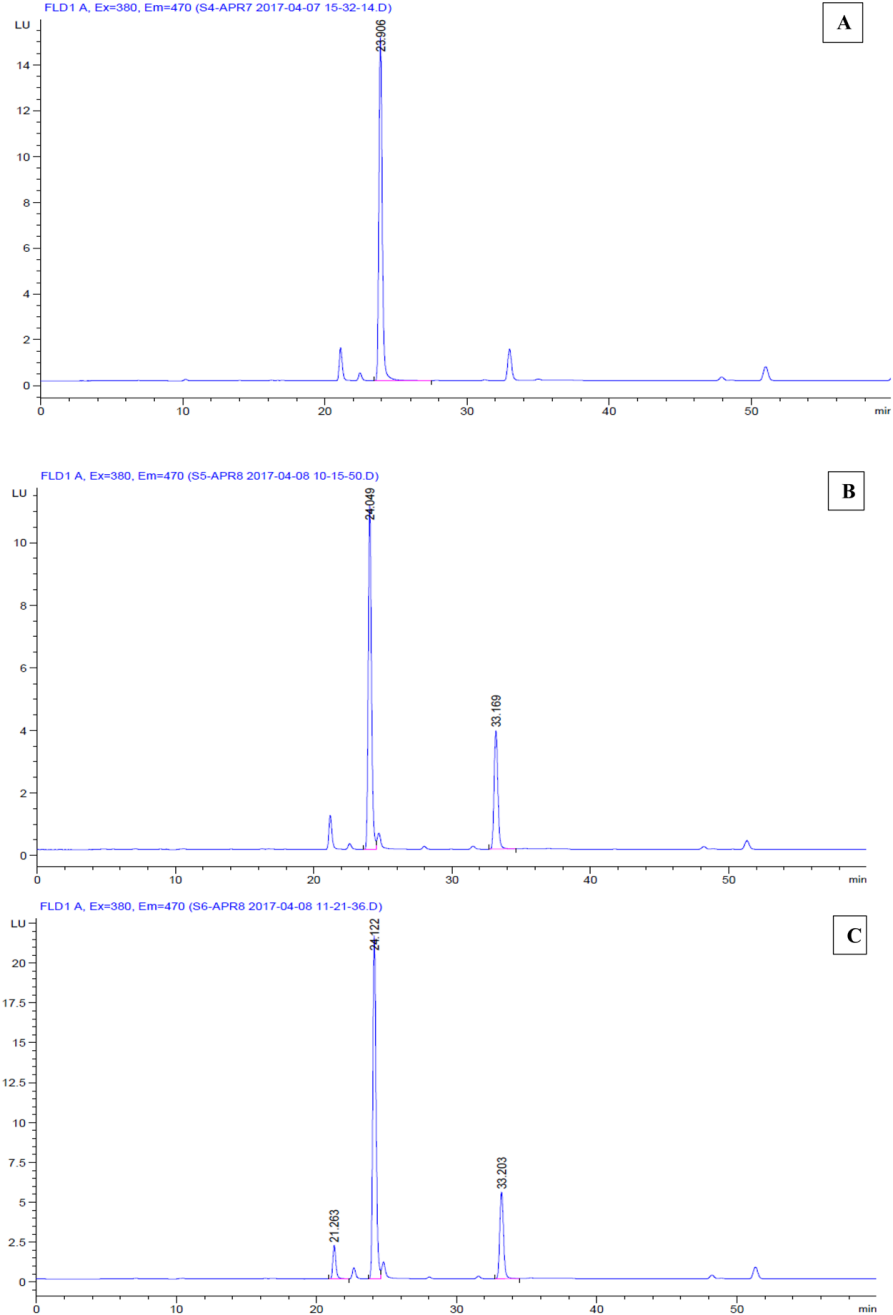
Chromatogram showing the expression of PC4 in *Scenedesmus rotundus* on the treatment with Cd and Zn. (**A**) control; (**B**) - Cadmium (CdCl_2_); (**C**) - Zinc (ZnCl_2_).

**Figure 8:**
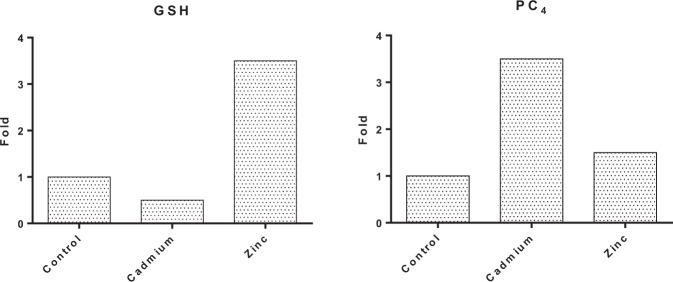
Effect of EC50 concentration of Cadmium and Zinc on fold change of thiol content in *Scenedesmus rotundus*. (**A**) GSH (**B**) PC4.

Figures 7 and 8 represents the variations in the levels of PC_4_ in *Scenedesmus rotundus* exposed to EC_50_ concentration of Cadmium and Zinc. While PC4 levels increased in both the treatments, induction of PC4 was strong in Cadmium treatment when compared to Zinc treated cells. The elevated levels of PC under Cd stress is also reported by Tukaj *et al*.^67^. Cd stress induces the synthesis of PC_2–4_. In the present study induction of PC_4_ was very strong in Cd treated *Scenedesmus rotundus* and the finding is in line with many other reports indicating strong induction of PCs in algal cells subjected to heavy metal stress.

Being in a highly reduced state under optimal conditions, shifting towards a more oxidized state in response to increased intracellular ROS availability and mechanisms that link such shifts to altered redox state, and therefore biological activity, of target proteins mark glutathione as a candidate transmitter of intracellular ROS signals. GSH content of the cell is crucial in determining the cells’ viability under heavy metal stress. Kov´aˇcik *et al*.^34^, reported that, old cultures that inherently had a lower GSH were not as viable as the new cultures that had a higher levels of the same. In the present study GSH levels showed different response to Cd and Zn treatments. While GSH pools were depleted upon Cd treatment, Zn treatment improved the levels of intracellular GSH, by nearly 5 fold when compared to the control. A correlation between falls in the GSH levels with simultaneous rise in PCs levels has been reported in many papers. Increased rates of PC synthesis require the formation of a complex between GSH and a heavy metal. Therefore it can be inferred that while *Scenedesmus rotundus* alleviates Cd toxicity via complexation to PC_4_ leading to sequestration and detoxification, Zn toxicity is handled via other detoxifying mechanisms involving the GSH Ascorbate cycle, as high levels of GSH are persistent in cells exposed to Zn. Thus it can be reiterated that the optimal levels of GSH are crucial given its role in ROS scavenging and antioxidant activity and also confirming its role as a precursor to PC synthesis.

Wang *et al*.^23^, reported decrease in the GSH content and increased PC synthesis upon exposure of cells to As (II) and As(V), under phosphate limitation, while Kov´aˇcik *et al*.^28^, reported sharp increase in PC content induced by Cd accompanied by a significant decrease in the GSH content in the new cultures under short term exposure. On the contrary, Jacinto *et al*.^68^, reported increased peaks of Zn-GSH and Hg-PCs complexes supported that increased PC synthesis might is favored by the increased GSH levels. While many studies report an inverse correlation between GSH and PC content in cells exposed to different heavy metals, few studies report direct correlation. Exposure to Pb increased the levels of GSH and cysteine and hence increased activity of phytochelatin synthase and induction of PC_5_ in addition to PC_2–4_^69^. In the present study, while *Scenedesmus rotundus* responded to Cd stress by using up the GSH pool towards synthesis of PC4 leading to sequestration for detoxification, the response to Zn was skewed towards retention of GSH pools and increase in activity of antioxidant enzymes.

### Effect of Cd and Zn on algal cell wall morphology

The cell wall is a complex structure composed of cellulose and polysaccharides, cross-linked with structural proteins. The cell wall represents a physical barrier against the entry of heavy metals into the symplastic compartment. Cell wall polysaccharides play a crucial role in heavy metal binding and accumulation in the cell wall^70,71^. One of the advantages of precipitating metals within the cell wall is their strong metabolic inactivation^72^.

**Figure 9:**
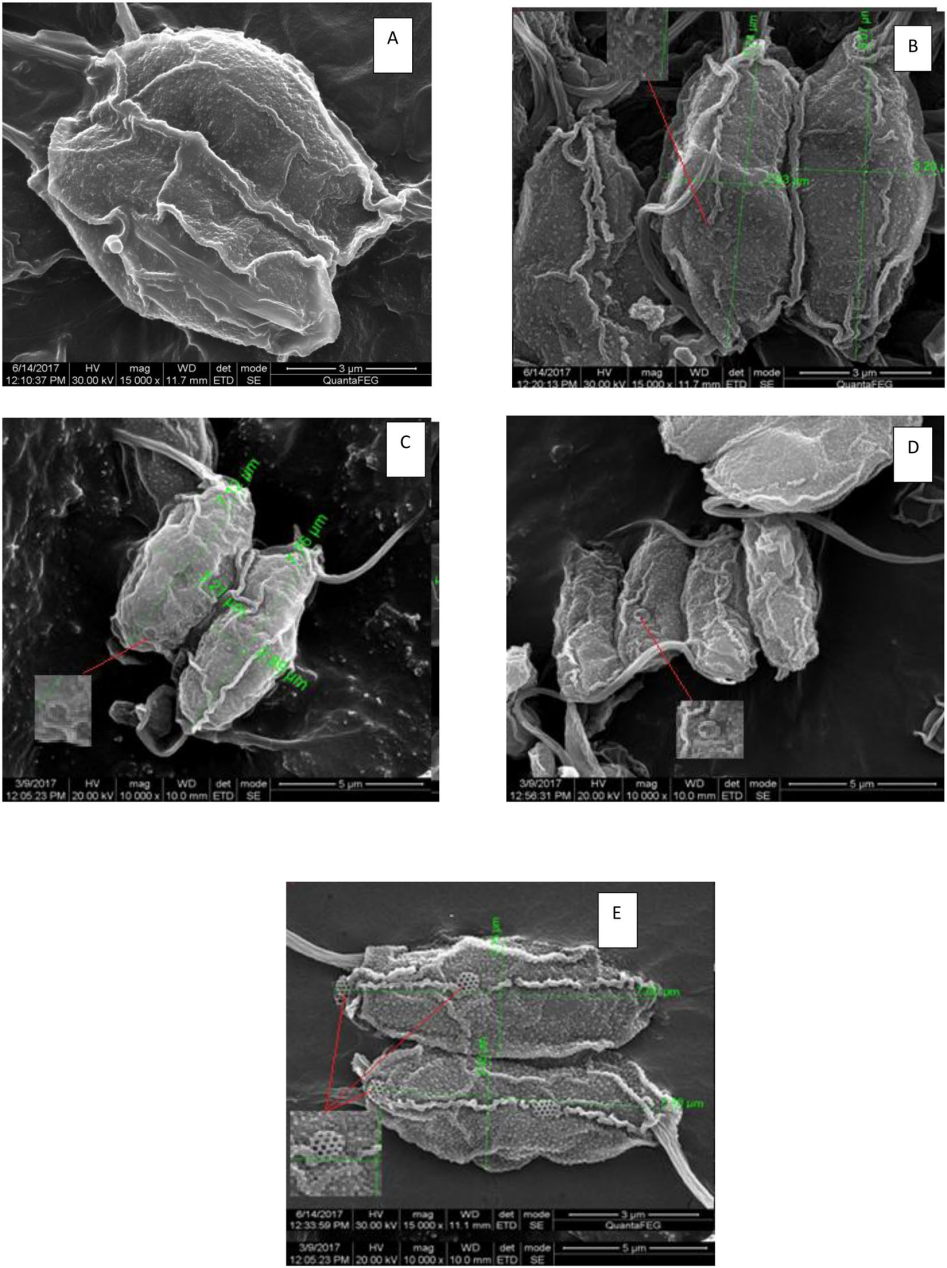
Scanning Electron Micrographs depicting structural changes in cell wall of *Senedesmus rotundus* treated with EC50 of Cadmium at (**A**) pH 6.8 (Control) (**B**) pH 5 (**C**) pH 8 (**D**) pH 10 (**E**) pH 12.

**Figure 10:**
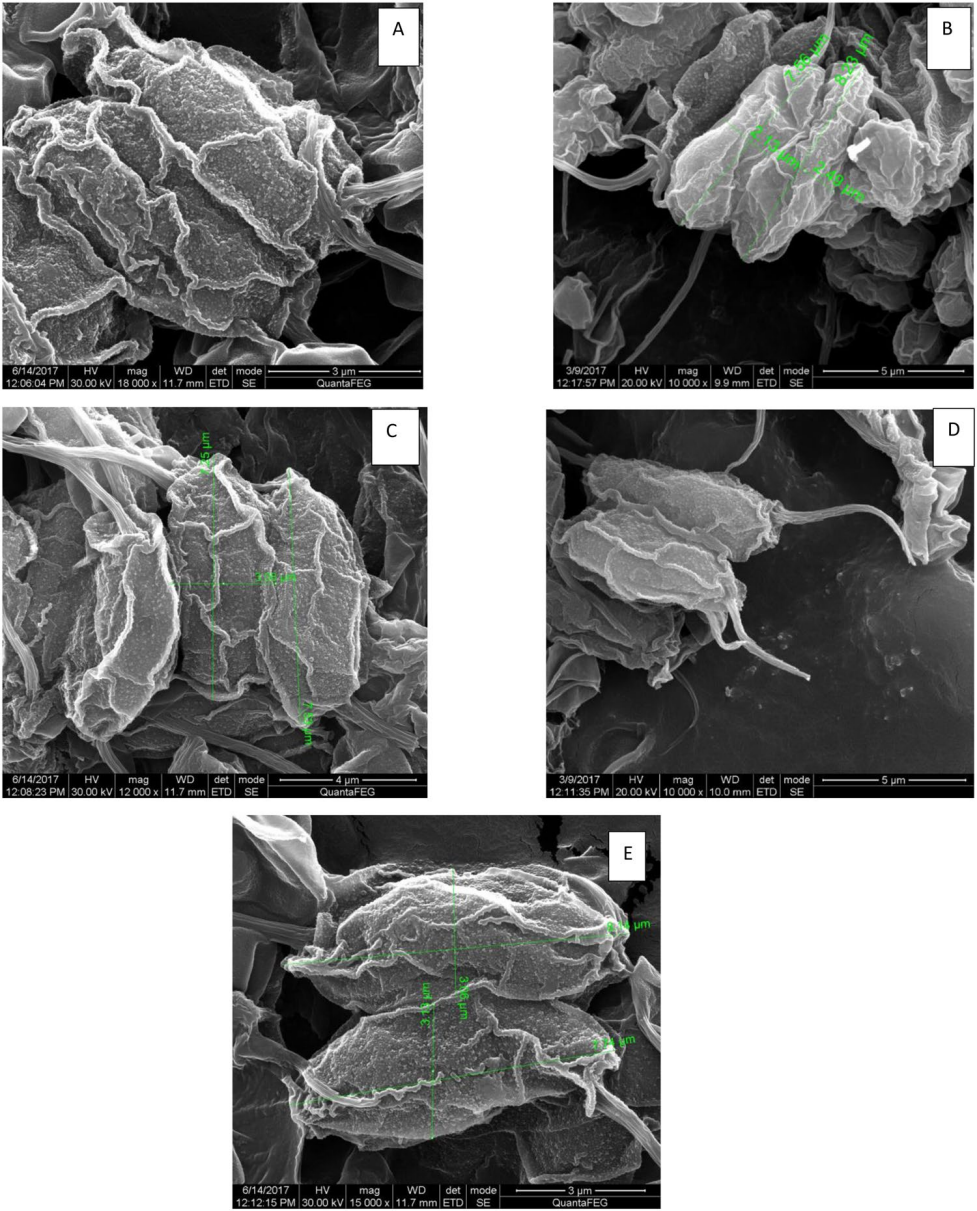
Scanning Electron Micrographs depicting structural changes in cell wall of *Scenedesmus rotundus* treated with EC50 of Zinc at (**A**) pH 6.8 (Control) (**B**) pH 5 (**C**) pH 8 (**D**) pH 10 (**E**) pH 12.

Figures 9 and 10 depict the scanning electron micrographs of *Scenedesmus rotundus* grown in different pH at EC_50_ concentrations of Cd and Zn. Marked changes in the architecture of the cell wall were observed in both the treatments. This is the first report on effect of heavy metals on the structural modifications of the cell wall in *Scenedesmus* sp. in general and *Scenedesmus rotundus* in particular. One of the major changes observed is the appearance of granules on the entire cell surface of the Cd and Zn treated samples. Such granules were sparsely and uniformly distributed in the control cells and cells grown at pH 5, while the cells grown at pH of 8, 10 and 12, an increase in the granulation is reported. Further the degree of granulation increased in the order of 12 ˃ 10 ˃ 8 with in Cd treatments. Samadani *et al*.^73^, report an increase in cell granularity was observed in Cd exposed cells which the authours attributed to Cd-S containing molecule complexes. *Scenedesmus* treated with EC_50_ of Zinc at different pH showed marginal reduction in cell size, however did not show variation in granulation as compared to the control. Zinc being an essential trace metal, is probably internally sequestered, while the toxic non - essential Cd may be precipitated in the cell wall and a portion of it rendered metabolically inactive. Andosch *et al*.^74^, reported the link between compartmentalization of heavy metals and the toxicity of these metals. Based on ultrastructural details of cellular organelles they reported that Cd and Cr, which were less compartmentalized into intracellular organelles, were more toxic than Al, Zn, and Cu.

**Figure 11:**
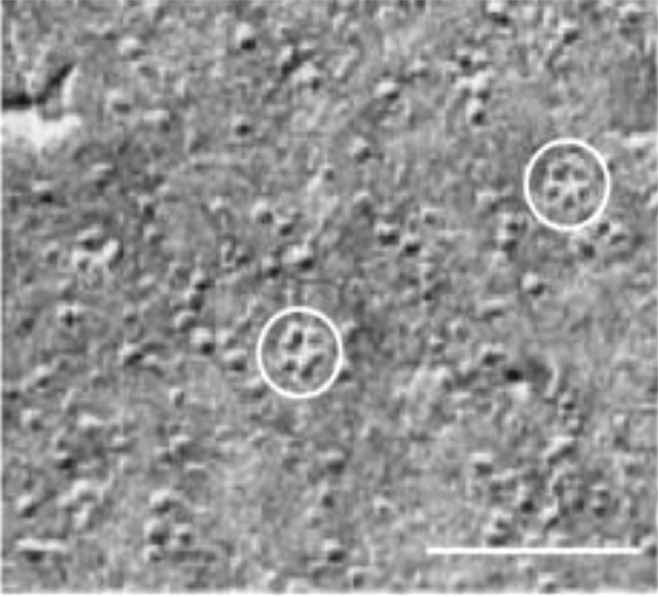
Cellulose synthase complexes (CSCs) in the plasma membrane of Arabidopsis. Scale bar = 100 nm. Adapted from Williamson *et al*. (2002). Copyright by Elsevier.

**Figure 12:**
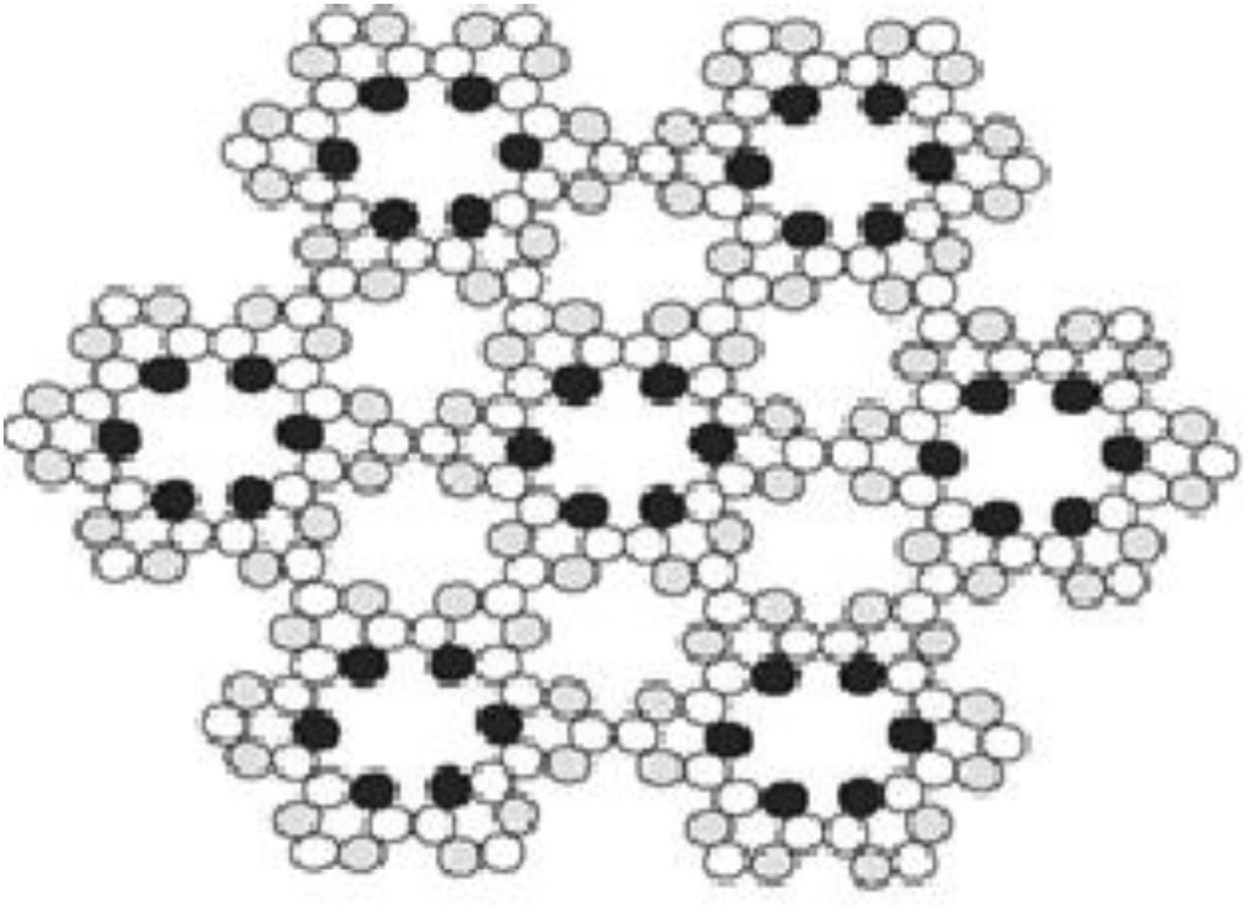
Schematic model structure of the cellulose synthases complex (rosettes) in higher plants. Reprinted with permission from Ding SY, Himmel ME. The maize primary cell wall microfibril: a new model derived from direct visualization, *J. Agric. Food Chem*, 54, 597–606, (2006). Copyright (2006) American Chemical Society.

Further structures of higher order resembling minute wheels are observed in Cd treated cells across all the pH, with the degree of order increasing with increased pH. The higher order structures as observed in Cd treated cells were completely absent in cells exposed to EC_50_ concentration of Zinc. A comparison of these structures could be drawn to cellulose synthase complexes in the plasma membrane of Arabidopsis^75,76^ (Figs 11 and 12). Do these represent synthetic machinery for formation of new wall material in *Scenedesmus rotundus* exposed to Cd stress or are these structures associated with the efflux of the metal has to be investigated to understand the functional significance of these structures with respect to alleviating metal toxicity in general and Cd tolerance in particular.
